# Guideline adherence in febrile children below 3 months visiting European Emergency Departments: an observational multicenter study

**DOI:** 10.1007/s00431-022-04606-5

**Published:** 2022-09-30

**Authors:** Chantal D. Tan, Eline E. P. L. van der Walle, Clementien L. Vermont, Ulrich von Both, Enitan D. Carrol, Irini Eleftheriou, Marieke Emonts, Michiel van der Flier, Ronald de Groot, Jethro Herberg, Benno Kohlmaier, Michael Levin, Emma Lim, Ian K. Maconochie, Federico Martinon-Torres, Ruud G. Nijman, Marko Pokorn, Irene Rivero-Calle, Maria Tsolia, Shunmay Yeung, Werner Zenz, Dace Zavadska, Henriëtte A. Moll, PERFORM consortium (Personalised Risk assessment in febrile children to optimize Real-life Management across the European Union), Michael Levin, Aubrey Cunnington, Tisham De, Jethro Herberg, Myrsini Kaforou, Victoria Wright, Lucas Baumard, Evangelos Bellos, Giselle D’Souza, Rachel Galassini, Dominic Habgood-Coote, Shea Hamilton, Clive Hoggart, Sara Hourmat, Heather Jackson, Ian Maconochie, Stephanie Menikou, Naomi Lin, Samuel Nichols, Ruud Nijman, Ivonne Pena Paz, Priyen Shah, Ching-Fen Shen, Ortensia Vito, Clare Wilson, Amina Abdulla, Ladan Ali, Sarah Darnell, Rikke Jorgensen, Sobia Mustafa, Salina Persand, Molly Stevens, Eunjung Kim, Benjamin Pierce, Katy Fidler, Julia Dudley, Vivien Richmond, Emma Tavliavini, Ching-Chuan Liu, Shih-Min Wang, Federico Martinón-Torres, Antonio Salas, Fernando Álvez González, Cristina Balo Farto, Ruth Barral-Arca, María Barreiro Castro, Xabier Bello, Mirian Ben García, Sandra Carnota, Miriam Cebey-López, María José Curras-Tuala, Carlos Durán Suárez, Luisa García Vicente, Alberto Gómez-Carballa, Jose Gómez Rial, Pilar Leboráns Iglesias, Federico Martinón-Torres, Nazareth Martinón-Torres, José María Martinón Sánchez, Belén Mosquera Pérez, Jacobo Pardo-Seco, Lidia Piñeiro Rodríguez, Sara Pischedda, Sara Rey Vázquez, Irene Rivero Calle, Carmen Rodríguez-Tenreiro, Lorenzo Redondo-Collazo, Miguel Sadiki Ora, Antonio Salas, Sonia Serén Fernández, Cristina Serén Trasorras, Marisol Vilas Iglesias, Dace Zavadska, Anda Balode, Arta Bārzdiņa, Dārta Deksne, Dace Gardovska, Dagne Grāvele, Ilze Grope, Anija Meiere, Ieva Nokalna, Jana Pavāre, Zanda Pučuka, Katrīna Selecka, Aleksandra Sidorova, Dace Svile, Urzula Nora Urbāne, Effua Usuf, Kalifa Bojang, Syed M. A. Zaman, Fatou Secka, Suzanne Anderson, Anna Roca, Isatou Sarr, Momodou Saidykhan, Saffiatou Darboe, Samba Ceesay, Umberto D’alessandro, Henriëtte A. Moll, Dorine M. Borensztajn, Nienke N. Hagedoorn, Chantal Tan, Clementien L. Vermont, Joany Zachariasse, W. Dik, Philipp Agyeman, Luregn J. Schlapbach, Christoph Aebi, Verena Wyss, Mariama Usman, Philipp Agyeman, Luregn J. Schlapbach, Eric Giannoni, Martin Stocker, Klara M. Posfay-Barbe, Ulrich Heininger, Sara Bernhard-Stirnemann, Anita Niederer-Loher, Christian Kahlert, Giancarlo Natalucci, Christa Relly, Thomas Riedel, Christoph Aebi, Christoph Berger, Enitan D. Carrol, Stéphane Paulus, Elizabeth Cocklin, Rebecca Jennings, Joanne Johnston, Simon Leigh, Karen Newall, Sam Romaine, Maria Tsolia, Irini Eleftheriou, Maria Tambouratzi, Antonis Marmarinos, Marietta Xagorari, Kelly Syggelou, Colin Fink, Marie Voice, Leo Calvo-Bado, Werner Zenz, Benno Kohlmaier, Nina A. Schweintzger, Manfred G. Sagmeister, Daniela S. Kohlfürst, Christoph Zurl, Alexander Binder, Susanne Hösele, Manuel Leitner, Lena Pölz, Glorija Rajic, Sebastian Bauchinger, Hinrich Baumgart, Martin Benesch, Astrid Ceolotto, Ernst Eber, Siegfried Gallistl, Gunther Gores, Harald Haidl, Almuthe Hauer, Christa Hude, Markus Keldorfer, Larissa Krenn, Heidemarie Pilch, Andreas Pfleger, Klaus Pfurtscheller, Gudrun Nordberg, Tobias Niedrist, Siegfried Rödl, Andrea Skrabl-Baumgartner, Matthias Sperl, Laura Stampfer, Volker Strenger, Holger Till, Andreas Trobisch, Sabine Löffler, Shunmay Yeung, Juan Emmanuel Dewez, Martin Hibberd, David Bath, Alec Miners, Ruud Nijman, Catherine Wedderburn, Anne Meierford, Baptiste Leurent, Ronald de Groot, Michiel van der Flier, Marien I. de Jonge, Koen van Aerde, Wynand Alkema, Bryan van den Broek, Jolein Gloerich, Alain J. van Gool, Stefanie Henriet, Martijn Huijnen, Ria Philipsen, Esther Willems, G.P.J.M. Gerrits, M. van Leur, J. Heidema, L. de Haan, C.J. Miedema, C. Neeleman, C.C. Obihara, G.A. Tramper-Stranders, Andrew J. Pollard, Rama Kandasamy, Stéphane Paulus, Michael J. Carter, Daniel O’Connor, Sagida Bibi, Dominic F. Kelly, Meeru Gurung, Stephen Thorson, Imran Ansari, David R. Murdoch, Shrijana Shrestha, Zoe Oliver, Marieke Emonts, Emma Lim, Lucille Valentine, Karen Allen, Kathryn Bell, Adora Chan, Stephen Crulley, Kirsty Devin, Daniel Fabian, Sharon King, Paul McAlinden, Sam McDonald, Anne McDonnell, Ailsa Pickering, Evelyn Thomson, Amanda Wood, Diane Wallia, Phil Woodsford, Frances Baxter, Ashley Bell, Mathew Rhodes, Rachel Agbeko, Christine Mackerness, Bryan Baas, Lieke Kloosterhuis, Wilma Oosthoek, Tasnim Arif, Joshua Bennet, Kalvin Collings, Ilona van der Giessen, Alex Martin, Aqeela Rashid, Emily Rowlands, Gabriella de Vries, Fabian van der Velden, Lucille Valentine, Mike Martin, Ravi Mistry, Ulrich von Both, Laura Kolberg, Manuela Zwerenz, Judith Buschbeck, Christoph Bidlingmaier, Vera Binder, Katharina Danhauser, Nikolaus Haas, Matthias Griese, Tobias Feuchtinger, Julia Keil, Matthias Kappler, Eberhard Lurz, Georg Muench, Karl Reiter, Carola Schoen, François Mallet, Karen Brengel-Pesce, Alexandre Pachot, Marine Mommert, Marko Pokorn, Mojca Kolnik, Katarina Vincek, Tina Plankar Srovin, Natalija Bahovec, Petra Prunk, Veronika Osterman, Tanja Avramoska, Taco Kuijpers, Ilse Jongerius, J.M. van den Berg, D. Schonenberg, A.M. Barendregt, D. Pajkrt, M. van der Kuip, A.M. van Furth, Evelien Sprenkeler, Judith Zandstra, G. van Mierlo, J. Geissler

**Affiliations:** 1 Department of General Paediatrics, Erasmus MC-Sophia Children’s Hospital, P.O. Box 2060, 3000 CB Rotterdam, the Netherlands; 2 Section of Paediatric Infectious Diseases, Imperial College, London, UK; 3 Rīgas Stradiņa universitāte, Department of Paediatrics, Children Clinical University Hospital, Riga, Latvia; 4 Division of Paediatric Infectious Diseases, Dr. Von Hauner Children’s Hospital, University Hospital, Ludwig-Maximilians-University Munich, Munich, Germany; 5 German Centre for Infection Research, DZIF, Partner Site Munich, Munich, Germany; 6 Institute of Infection, Veterinary and Ecological Sciences Liverpool, University of Liverpool, Liverpool, UK; 7 Alder Hey Children’s NHS Foundation Trust, Liverpool, UK; 8 Second Department of Paediatrics, National and Kapodistrian University of Athens, P. and A. Kyriakou Children’s Hospital, Athens, Greece; 9 Paediatric Immunology, Infectious Diseases & Allergy, Great North Children’s Hospital, Newcastle Upon Tyne Hospitals NHS Foundation Trust, Newcastle upon Tyne, UK; 10 Translational and Clinical Research Institute, Newcastle University, Newcastle upon Tyne, UK; 11 Section of Paediatric Infectious Diseases, Laboratory of Medical Immunology, Radboud Center for Infectious Diseases, Radboud Institute for Molecular Life Sciences, RadboudUMC, Nijmegen, the Netherlands; 12 Paediatric Infectious Diseases and Immunology, Amalia Children’s Hospital, RadboudUMC, Nijmegen, the Netherlands; 13 Paediatric Infectious Diseases and Immunology, Wilhelmina Children’s Hospital, University Medical Centre Utrecht, Utrecht, The Netherlands; 14 Department of General Paediatrics, Medical University of Graz, Graz, Austria; 15 Paediatric Emergency Medicine, Imperial College Healthcare Trust NHS, London, UK; 16 Vaccines, Infections and Paediatrics Research Group (GENVIP), Hospital Clínico Universitario de Santiago de Compostela. Genetics, Santiago de Compostela, Spain; 17 Department of Infectious Diseases and Faculty of Medicine, University Medical Centre Ljubljana, University of Ljubljana, Ljubljana, Slovenia; 18 Department of Paediatric Infectious Diseases and Immunology, Erasmus MC-Sophia Children’s Hospital, Rotterdam, the Netherlands; 19 NIHR Newcastle Biomedical Research Centre Based at Newcastle Upon Tyne Hospitals NHS Trust and Newcastle University, Westgate Rd, Newcastle upon Tyne, NE4 5PL UK; 20 London School of Hygiene and Tropical Medicine, Faculty of Tropical and Infectious Disease, London, UK; 21 Department of Medicine, Population Health Sciences Institute, Newcastle University, Newcastle upon Tyne, UK

**Keywords:** Fever, Children, Pediatrics, Guideline, Emergency care

## Abstract

Febrile children below 3 months have a higher risk of serious bacterial infections, which often leads to extensive diagnostics and treatment. There is practice variation in management due to differences in guidelines and their usage and adherence. We aimed to assess whether management in febrile children below 3 months attending European Emergency Departments (EDs) was according to the guidelines for fever. This study is part of the MOFICHE study, which is an observational multicenter study including routine data of febrile children (0–18 years) attending twelve EDs in eight European countries. In febrile children below 3 months (excluding bronchiolitis), we analyzed actual management compared to the guidelines for fever. Ten EDs applied the (adapted) NICE guideline, and two EDs applied local guidelines. Management included diagnostic tests, antibiotic treatment, and admission. We included 913 children with a median age of 1.7 months (IQR 1.0–2.3). Management per ED varied as follows: use of diagnostic tests 14–83%, antibiotic treatment 23–54%, admission 34–86%. Adherence to the guideline was 43% (374/868) for blood cultures, 29% (144/491) for lumbar punctures, 55% (270/492) for antibiotic prescriptions, and 67% (573/859) for admission. Full adherence to these four management components occurred in 15% (132/868, range 0–38%), partial adherence occurred in 56% (484/868, range 35–77%).

*Conclusion*: There is large practice variation in management. The guideline adherence was limited, but highest for admission which implies a cautious approach. Future studies should focus on guideline revision including new biomarkers in order to optimize management in young febrile children.

**Table:** 

**What is Known:** *• Febrile children below 3 months have a higher risk of serious bacterial infections, which often leads to extensive diagnostics and treatment.* *• There is practice variation in management of young febrile children due to differences in guidelines and their usage and adherence.*	
**What is New:** *• Full guideline adherence is limited, whereas partial guideline adherence is moderate in febrile children below 3 months across Europe.* *• Guideline revision including new biomarkers is needed to improve management in young febrile children.*

**Supplementary Information:**

The online version contains supplementary material available at 10.1007/s00431-022-04606-5.

## Introduction

Fever is a very common presenting symptom in children visiting the emergency department (ED), accounting for approximately 20% of all pediatric emergency visits [1–3]. It remains challenging to clinically distinguish the majority having viral illnesses from serious bacterial infections (SBIs) such as urinary tract infection, pneumonia, sepsis, or meningitis. On one hand, this often leads to extensive diagnostic testing, antibiotic prescription, high hospitalization rates, and medical costs [4–6]. On the other hand, delayed recognition and treatment of SBIs can lead to substantial morbidity and mortality [7].

Children below 3 months of age have a higher risk of SBI, namely 5–15%, compared to older children due to specific pathogens, their immature immune system, and absent or incomplete vaccinations [6, 8–11]. Therefore, the threshold for diagnostic testing, antibiotic treatment, and hospital admission is lower in these children. Almost all vaccination programs in Europe start at an age of 2 or 3 months with differences in immunization rates within and across European countries [12, 13].

Currently, several guidelines have been developed for management of febrile children below 3 months [14–16]. These guidelines are substantially overlapping, but there is practice variation in guideline usage and adherence [17–19]. Clinical practice guidelines (CPGs) were successful in safely reducing diagnostic tests, antibiotic treatment and hospital admission with an adjusted odds ratio of 0.30 after implementation of a CPG in 400 children below 2 months at an American ED [20]. The National Institute for Health and Care Excellence (NICE) guideline of fever in children under 5 years is predominantly used in Europe [14]. Management in children below 3 months is advised based on a combination of general appearance and biomarkers, such as C-reactive protein (CRP) and white blood cell (WBC) count. The aim of this study is to provide insight in management of febrile children below 3 months attending European EDs, and to assess adherence to available fever guidelines, in order to identify areas for improvement.

## Methods

### Study design and setting

This study is part of the MOFICHE study (Management and Outcome of Fever in children in Europe), which is embedded in the PERFORM project (Personalized Risk assessment in Febrile illness to Optimize Real-life Management across the European Union) [21]. The MOFICHE study is an observational multicenter study evaluating management and outcome of febrile children in twelve EDs across eight European countries (Austria, Germany, Greece, Latvia, the Netherlands *n* = 3, Slovenia, Spain, UK *n* = 3). The hospital characteristics are shown in Appendix A and described in a previous study [13]. Approval by the ethics committees of the participating hospitals was obtained. The need for informed consent was waived.

### Study population

Data of 38,480 children with fever (≥ 38 ℃) at the ED or in three consecutive days before ED visit aged 0–18 years were collected between January 2017 and April 2018. For this study, only febrile children below 3 months of age were included. Children with comorbidities or missing data on management were excluded. Additionally, we excluded febrile children with bronchiolitis caused by respiratory syncytial virus for the analysis concerning guideline adherence, since management differs in these children [22].

### Data collection

Data were routinely collected from electronic patient records in a standardized pseudo-anonymized database for at least 1 year to include all four seasons, wherein inclusion varied from 1 week per month to the whole month per ED (Appendix A). The collected data included patient characteristics (age, gender, comorbidity (chronic condition expected to last at least 1 year [23]), presenting symptoms), disease severity (triage urgency, type of referral, vital signs), diagnostic tests (laboratory tests, imaging), antibiotic treatment, admission, focus, and cause of infection. Presenting symptoms were categorized into four groups: neurological (focal neurological signs or meningeal signs), respiratory (coughing or other signs of respiratory infections), gastrointestinal (vomiting or diarrhea), and other (non-specified)). Age specific cutoff values from Advanced Pediatric Life Support were used to categorize the vital signs into tachycardia, tachypnea, and hypoxia [24]. Increased work of breathing was defined as the occurrence of chest wall retractions, nasal flaring, grunting, or apneas. Focus of infection was categorized into upper respiratory tract, lower respiratory tract, gastrointestinal tract, urinary tract, flu-like illness or childhood exanthemas, soft tissue, skin or musculoskeletal infection, sepsis or meningitis, and other (e.g., undifferentiated fever, inflammatory illness). Lastly, the cause of infection was determined by the research team using a previously published phenotyping algorithm [5, 25] (Appendix B). It combines clinical data and diagnostic results to assign the presumed cause of infection. The cause of infection was categorized into definite bacterial, probable bacterial, bacterial syndrome, unknown bacterial or viral, definite viral, probable viral, viral syndrome, and other (e.g., inflammatory illness). SBI was defined as a lower respiratory tract infection, gastrointestinal infection, urinary tract infection, sepsis, meningitis, or musculoskeletal infection in combination with a probable or definite bacterial cause.

### Outcome measures

The main outcome of this study is management, which is divided into diagnostic tests, antibiotic treatment, and hospital admission. Diagnostic tests are categorized into simple and advanced diagnostic tests, where simple is considered less invasive, and advanced is considered more invasive for the child. Simple diagnostic tests included CRP, WBC count, Procalcitonin (PCT), urinalysis, urine culture, ultrasound, chest X-ray, respiratory test, or sputum culture. Blood culture, lumbar puncture, CT scan, or MRI scan are considered advanced diagnostic tests. If patients underwent both simple and advanced diagnostic tests, they were classified as advanced. Data on antibiotic prescription as well as group of antibiotics (narrow or broad spectrum) and route of administration (oral or parenteral) were collected. Narrow spectrum antibiotics included penicillins and first-generation cephalosporins. Broad spectrum antibiotics included penicillins with beta-lactamase inhibitor combinations, macrolides, aminoglycoside, glycopeptides, and second- and third-generation cephalosporins [5]. Children were discharged home or admitted to the pediatric ward (< 24 h or > 24 h) or pediatric intensive care unit.

The principal investigator of each hospital was asked which guideline for fever was available at their ED. A distinction was made into NICE, national or local guidelines for fever. Additionally, they were asked whether their guideline for fever was based on the NICE guideline for fever and specifically if the guideline contained the same diagnostic and therapeutic strategies as the NICE guideline [14], shown in Table 1. The four most important components of management according to the guidelines were compared with actual management performed in clinical practice at the ED: blood culture, lumbar puncture, antibiotic treatment, and hospital admission. Full adherence was defined as having blood cultures, lumbar punctures, antibiotic treatment, and hospital admission, all according to the recommendations of the available guideline for fever. Partial adherence was defined as following one to three of these four components. Children were classified as non-adherent when none of the four components was performed according to the guideline.

### Data analysis

Firstly, descriptive statistics were used to describe clinical characteristics and management. The range per ED was shown as well to show the variability. Additionally, management was shown stratified for EDs with high and low prevalence of SBI. The cutoff value for a high prevalence of SBI was 12%, which was determined by the prevalence of SBI in our study population. Secondly, management performed including blood culture, lumbar puncture, antibiotic treatment, and hospital admission of children below 3 months was compared to the available guideline for fever per ED. Subsequently, we performed subgroup analyses in children below 1 month and children 1–3 months. Thirdly, we analyzed the three adherence groups (full, partial, non) stratified for working diagnosis. Working diagnosis was categorized into presumed bacterial (definite bacterial, probable bacterial, bacterial syndrome), presumed viral (definite viral, probable viral, viral syndrome), and unknown bacterial or viral or other cause (Appendix B). *P*-values < 0.05 were considered statistically significant. Data were statistically analyzed using IBM SPSS software version 25.

## Results

### Patient characteristics and management

The population for analysis consisted of 913/38,480 (2%) febrile children below the age of 3 months. The median age was 1.7 months (IQR, 1.0–2.3) and the majority were boys (58%). Fifty-four percent of the children were referred by a physician and triaged as intermediate/high urgent (53%). The respiratory tract was the most common focus of infection, and the majority had a viral cause of infection (Table 2) (Appendix C). The causative pathogens stratified for bacteria and viruses are shown in Appendix D. Management in children below 3 months is also shown in Table 2. Only simple diagnostic tests were performed in 37%, of which CRP and WBC were performed most frequently (75% and 73%). Advanced diagnostics tests were performed in 44%, of which blood cultures were performed in 43% and lumbar punctures in 22%. Antibiotics were prescribed in 41% of which the majority received parenteral (92%) and broad spectrum antibiotics (89%). Sixty-eight percent of the children were admitted, of which 80% were admitted more than 24 h. Management per ED varied as shown in Table 2 and management was not associated with the prevalence of SBI at the ED as shown in Appendix E.

**Table 1:** Overview guidelines for fever below 3 months and recommendations

Guideline	Blood culture	Lumbar puncture	Antibiotic treatment	Hospital admission
NICE guideline of fever in under 5 s (*N* = 1 ED) or adapted version with same diagnostic and therapeutic strategies (*N* = 9 EDs)	Always in children < 3 months	All children < 1 month All children aged 1–3 months who appear ill or with abnormal WBC*	Indications: all children < 1 month, all children aged 1–3 months who appear ill or with abnormal WBC* Choice of antibiotics: third generation cephalosporin + ampicillin or amoxicillin; both intravenous Duration: depending on culture results	Always in children < 3 months
Liverpool, UK (local guideline)	Always in children < 3 months	Always in children < 3 months	Indications: all febrile children < 3 months Choice of antibiotics: third generation cephalosporin + amoxicillin; both intravenous Duration: review antibiotics after 48 h	Always in children < 3 months
Graz, Austria (local guideline)	Always in children < 3 months	In all children < 3 months, except for well appearance, unremarkable WBC* and CRP^a^	Indications: all children < 1 month, children aged 1–3 months who appeared ill, or with abnormal WBC*, children that were unarousable, in shock or showing signs of meningococcal disease Choice of antibiotics: no recommendations Duration: no recommendations	Yes, in all children < 3 months, except for children > 28 days with well appearance, unremarkable WBC* and CRP^a^

*Missing or WBC < 5 or > 15 × 10^9/L

^a^Missing or CRP < 20 mg/L

**Table 2:** Patient characteristics and management (*N* = 913)

	**Febrile Children below 3 months**	**Range per ED** (%)	**Missing**
**Age** (months) ^a^	1·7 (1·0–2·3)	0·9–2·3	-
**Gender** (boys)	526 (58)	(42–71)	-
**Referred**	495 (54)	(14–100)	10 (1)
**Triage urgency**			56 (6)
-Low	371 (41)	(4–96)	
-Intermediate/high	486 (53)	(4–87)	
**Ill appearance**	214 (23)	(4–57)	69 (8)
**Presenting symptom** ^b^			
-Neurological	24 (3)	(0–13)	94 (10)
-Respiratory	438 (48)	(21–83)	143 (16)
-Gastrointestinal	201 (22)	(8–37)	137 (15)
-Other	369 (40)	(9–62)	0 (0)
**Vital signs**			
Tachycardia	220 (24)	(6–46)	73 (8)
Tachypnea	176 (19)	(4–29)	193 (21)
Hypoxia	35 (4)	(0–9)	103 (11)
Temperature (°C) ^a^	37·6 (37·0–38·2)	37·1–38·5	68 (7)
**Duration of fever** (days) ^a^	0·5 (0·5–1·5)	0·5–0·5	88 (10)
**Increased work of breathing**	103 (11)	(1–21)	153 (17)
**Focus of infection**			-
-Upper respiratory tract	229 (25)	(3–50)	
-Lower respiratory tract	138 (15)	(0–30)	
-Gastrointestinal tract	54 (6)	(0–18)	
-Urinary tract	94 (10)	(4–24)	
-Flu like illness or exanthema	35 (4)	(1–10)	
-Soft tissue, skin or musculoskeletal	22 (2)	(0–7)	
-Sepsis/meningitis	81 (9)	(0–36)	
-Other	260 (29)	(9–46)	
**Diagnostic tests**			-
-**No diagnostic tests**	**179 (20)**	**(0**–**34)**	
-**Only simple diagnostic tests **^**c**^	**333 (37)**	**(16**–**83)**	
-*CRP*	*251 (75)*	*(27*–*100)*	
-*White blood cell count*	*242 (73)*	*(32*–*100)*	
-*PCT*	*10 (3)*	*(0*–*33)*	
-*Urinalysis*	*190 (57)*	*(23*–*80)*	
-*Urine culture*	*68 (20)*	*(0*–*67)*	
-*Ultrasound*	*26 (8)*	*(0*–*16)*	
-*Chest X-ray*	*46 (14)*	*(0*–*37)*	
-*Respiratory test/sputum culture*	*81 (24)*	*(7*–*60)*	
-**Advanced diagnostic tests **^**c**^	**401 (44)**	**(14**–**67)**	
-*CRP*	*394 (98)*	*(88*–*100)*	
-*White blood cell count*	*390 (97)*	*(81*–*100)*	
-*PCT*	*50 (13)*	*(0*–*92)*	
-*Urinalysis*	*293 (73)*	*(50*–*98)*	
-*Urine culture*	*288 (72)*	*(24*–*96)*	
-*Ultrasound*	*61 (15)*	*(0*–*31)*	
-*Chest X-ray*	*65 (16)*	*(0*–*50)*	
-*Respiratory test/sputum culture*	*152 (38)*	*(0*–*81)*	
-*Blood culture*	*392 (98)*	*(13*–*67)*	
-*Lumbar puncture*	*197 (49)*	*(0*–*38)*	
-*CT scan*	*3 (1)*	*(0*–*4)*	
-*MRI scan*	*0 (0)*	*(0)*	
**Antibiotic treatment **^cd^ -Oral -Parenteral -Narrow spectrum -Broad spectrum	**374 (41)** 27 (7) 345 (92) 33 (9) 334 (89)	**(23**–**54)** (0–45) (55–100) (0–19) (81–100)	-
**Hospital admission **^ce^ -Hospital admission > 24 h ^d^ -Admission to PICU	**621 (68)** 498 (80) 11 (2)	**(34–86)** (17–74) (0–7)	-

Absolute numbers and percentages (%) are shown

^a^Median and interquartile range (IQR) are shown; range per ED: lowest and highest median are shown

^b^It is possible to have more than one presenting symptom. If children had no neurological, respiratory, and gastrointestinal symptoms, they were classified as other

^c^Percentages per subcategory are based on the total number of children per management category

^d^Missing data on route of administration antibiotics and type of antibiotics (0·5–2%), and duration of hospital admission (3%)

^e^No children died in this cohort

### Guideline adherence

**Fig. 1:**
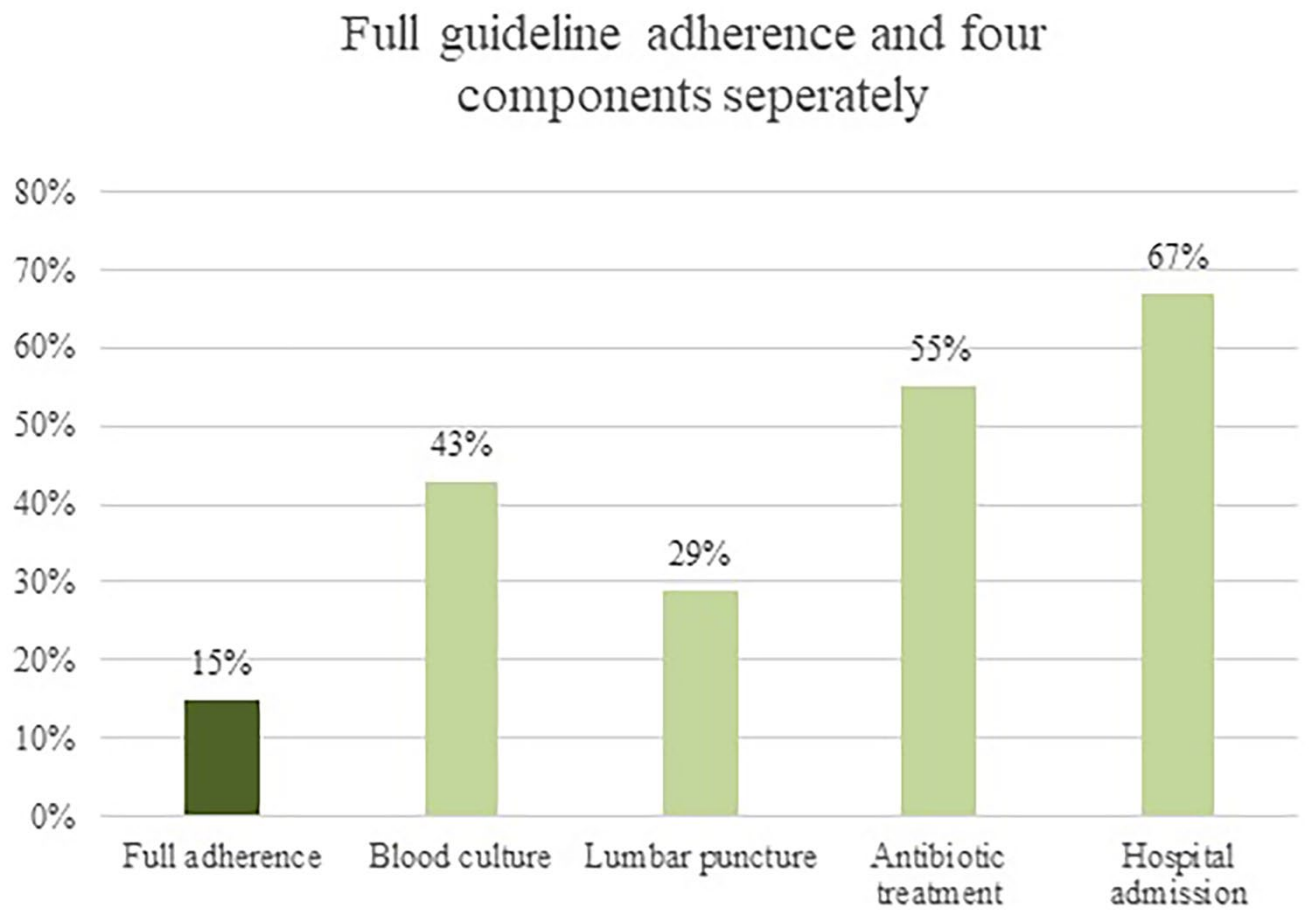
Guideline adherence in children below 3 months (*N* = 868)

**Fig. 2:**
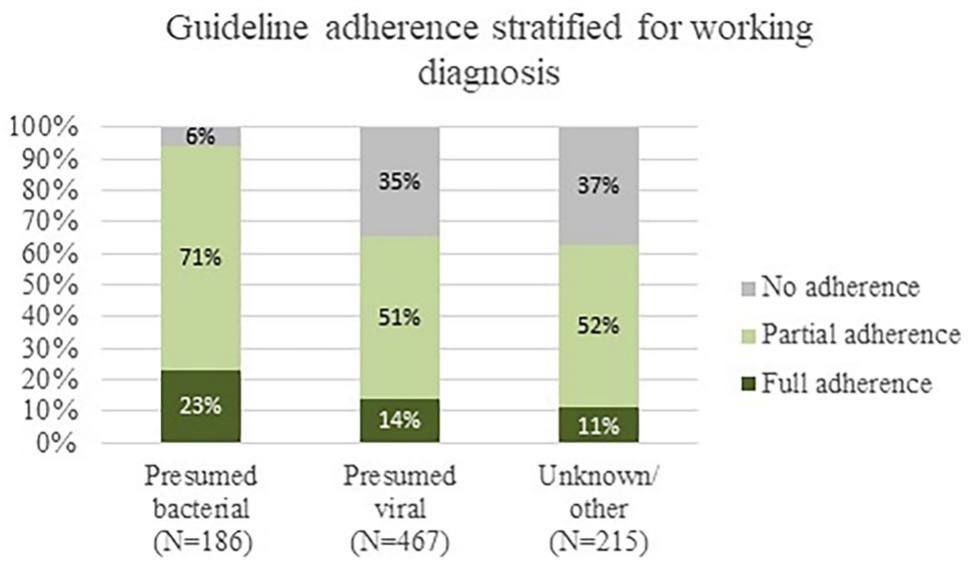
Guideline adherence stratified for working diagnosis (*N* = 868)

Guideline adherence in febrile children (excluding bronchiolitis) below 3 months (*N* = 868) is shown in Fig. 1 and the range per ED is shown in Appendix F. Guideline adherence varied as follows: blood cultures were obtained in 43% (374/868, range 13–67%), lumbar punctures in 29% (144/491, range 0–62%), antibiotics were prescribed in 55% (270/492, range 33–80%), and 67% (573/859, range 31–91%) were admitted. Full adherence to the guideline occurred in 15% (132/868, range 0–38%), partial adherence to the guideline occurred in 56% (484/868, range 35–77%), and no adherence occurred in 29% (252/868, range 13–45%). The majority fulfilled the criteria of partial adherence since these children were admitted according to the guideline (441/484, 91%). The three adherence groups stratified for working diagnosis are shown in Fig. 2. Twenty-one percent (186/868) of the children had a presumed bacterial infection, 54% (467/868) had a presumed viral infection, and 25% (215/868) had an unknown or other infection. In children with a presumed bacterial infection, full adherence occurred in 23% (42/186), partial adherence in 71% (133/186), and 90% (167/186) were admitted. In children with a presumed viral infection, full adherence occurred in 14% (66/467), partial adherence occurred in 51% (239/467), and 61% were admitted (283/467). Management and guideline adherence stratified for children below 1 month (*N* = 231) and 1 to 3 months (*N* = 682) is shown in Appendix G. Children below 1 month received more often advanced diagnostic tests (50% versus 42%), received antibiotic treatment more frequently (55% versus 36%), and were admitted more frequently (76% versus 65%) compared with children aged 1 to 3 months. Full adherence to the guideline in children below 1 month was 32% (71/223) compared with 10% (61/645) in children aged 1 to 3 months.

## Discussion

### Main findings

In this study, we examined management and guideline adherence in febrile children below 3 months, which covers 2% of the total pediatric population with fever attending twelve European EDs. Twelve percent of these children had a SBI which corresponds with previous literature where the percentage of SBI in children below 3 months varied between 5 and 15% [6, 8, 9]. There was large practice variation in management across the EDs, in which diagnostic tests ranged from 14 to 83%, antibiotic treatment ranged from 23 to 54%, and admission ranged from 34 to 86%. No association between settings with a high prevalence of SBI (> 12%) and more extensive management was found. Full guideline adherence was limited, namely 15% (132/868, range 0–38%), but partial guideline adherence was moderate 56% (484/868, range 35–77%), of which the majority (91%) were adherent to the admission component. In the subgroup analysis, we have seen that full adherence to the guideline occurred more often in children below 1 month compared with children 1–3 months (32% versus 10%). When we describe the four management components separately, guideline adherence varied as follows: a blood culture was obtained in 43%, a lumbar puncture in 29%, antibiotic treatment was given in 55%, and 67% were admitted. The high percentage of adherence for hospital admission (67%) could be interpreted as a cautious approach. The low adherence for lumbar punctures could be due to the physicians’ decision but also due to failure of lumbar punctures, which was described to occur in 38% of children below 3 months [26]. Additionally, in children with a presumed bacterial infection, full guideline adherence occurred in 23%, partial guideline adherence occurred in 71%, and 90% were admitted. In children with a presumed viral infection, full guideline adherence occurred in 14%, partial guideline adherence in 51%, and 61% was admitted.

### Strengths and limitations

To our knowledge, this is the first study to examine actual management and adherence to guidelines for fever in children below 3 months using a large European multicenter ED cohort. High-quality routine data was collected extensively, which made it possible to compare actual management (diagnostic tests, treatment, admission) with the management recommended by the guidelines for fever. There are some limitations as well. No data on follow-up was collected in this study, which made it difficult to interpret the outcome of guideline adherence. However, the majority were admitted and we used the phenotyping algorithm as proxy for the working diagnosis, which had a good performance in allocating a bacterial cause of infection [25]. Additionally, we had data on whether children died and this was not the case in our cohort. Participating EDs were part of large university or teaching hospitals, which might limit generalizability to general hospitals. However, an additional analysis examining management stratified for prevalence on SBI did not show any differences in management. The hypothesis that EDs would perform more extensive management when the proportion of children with SBI is high was not reflected in the results, which implies that differences in management across EDs do not appear to be related to the prevalence of SBI (Appendix E). Lastly, we defined adherence as management performed according to the guideline and non-adherence as management not performed as recommended by the guideline. However, there was a small proportion of children in whom extensive management was performed in whom it was not recommended by the guideline, but other factors might have led to these diagnostics and treatment. We did not allocate these cases as non-adherent.

### Implications for clinical practice

This study shows large practice variation in management across EDs and limited full adherence, but moderate partial adherence to guidelines for fever, which once again highlights that managing these febrile children below 3 months is challenging. On one hand, missing SBIs can lead to morbidity and even mortality. On the other hand, these children often receive extensive diagnostic testing and antibiotic treatment and are admitted leading to high medical costs and great impact on children and parents. The discussion on diagnostic uncertainty and hospitalization of febrile children below 3 months already started about 40 years ago, when a study by DeAngelis et al. showed that approximately 20% of hospitalized, febrile children below 2 months had complications due to diagnostic tests, antibiotic treatment, or the hospitalization itself [27]. Since then, many studies examined the use of guidelines and prediction models for the risk of SBI in young febrile children to reduce antibiotic treatment and hospital admission. The adherence to the guidelines for fever was limited in our study, which may raise the question whether our current guidelines are interpreted differently and probably too cautious. However, when children were not fully managed according to the guideline, most of them were admitted and this implies a cautious approach. Furthermore, management at the ED can be influenced by many factors, such as parental concern, physicians’ working experience, overcrowding, and nurses’ and physicians’ gut feeling. However, overall clinical impression of experienced nurses at the ED is not an accurate predictor of serious illness in children and clinician’s gut feeling is not predictive for diagnosing SBIs [28, 29]. We suggest to improve management of febrile children below 3 months by revising the guidelines, since physicians make different decisions regarding management than is recommended by the guidelines. Before revising the guidelines, it would be beneficial if physicians can substantiate their decision-making concerned management including blood culture, lumbar puncture, and antibiotic prescription in cases of febrile children below 3 months attending the ED. Additionally, as the final cause of infection was predominantly viral, there is room for improvement in management by reducing antibiotics and admission in this group, which contributes to lowering antimicrobial resistance and medical costs associated with admission. The American Academy of Pediatrics’ CPG for febrile children below 2 months recommend less extensive management based on age and well appearance but should be validated in a European cohort [30]. The proportion of full adherence to the guideline was higher for bacterial than for viral infections, which implies that the guidelines are contributing to the decision-making process. However, CPGs should be improved to guide decision-making, since none of several CPG’s studied demonstrated ideal performance characteristics in previous research [31]. Discovery and implementation of a new biomarker in the guidelines for young febrile children could improve the ability to make a better distinction between bacterial or viral infections. A promising biomarker in distinguishing bacterial and viral infections in febrile children is based on the RNA host response [32–34].

## Conclusion

There is large practice variation in management in febrile children below 3 months attending European EDs. Full guideline adherence was limited, but highest in children with a presumed bacterial infection. Partial adherence was moderate, with highest compliance for admission, which implies a cautious but expensive approach. Future studies should focus on guideline revision including new biomarkers in order to optimize management in young febrile children.

## Supplementary Information

Below is the link to the electronic supplementary material.

Supplementary file1 (PDF 742 KB)

## Abbreviations

CPG: Clinical practice guideline
CRP: C-reactive protein
ED: Emergency department
MOFICHE: Management and outcome of fever in children in Europe
NICE: The National Institute for Health and Care Excellence
PCT: Procalcitonin
PERFORM: Personalized risk assessment in febrile illness to optimize real-life management across the European Union
SBI: Serious bacterial infection
WBC: White blood cell count
