# Correction to: Guideline adherence in febrile children below 3 months visiting European Emergency Departments: an observational multicenter study

**DOI:** 10.1007/s00431-022-04664-9

**Published:** 2022-10-21

**Authors:** Chantal D. Tan, Eline E. P. L. van der Walle, Clementien L. Vermont, Ulrich von Both, Enitan D. Carrol, Irini Eleftheriou, Marieke Emonts, Michiel van der Flier, Ronald de Groot, Jethro Herberg, Benno Kohlmaier, Michael Levin, Emma Lim, Ian K. Maconochie, Federico Martinon-Torres, Ruud G. Nijman, Marko Pokorn, Irene Rivero-Calle, Maria Tsolia, Shunmay Yeung, Werner Zenz, Dace Zavadska, Henriëtte A. Moll, PERFORM consortium (Personalised Risk assessment in febrile children to optimize Real-life Management across the European Union)

**Affiliations:** 1 Department of General Paediatrics, Erasmus MC-Sophia Children’s Hospital, P.O. Box 2060, 3000 CB Rotterdam, the Netherlands; 2 Department of Paediatric Infectious Diseases and Immunology, Erasmus MC-Sophia Children’s Hospital, Rotterdam, the Netherlands; 3 Division of Paediatric Infectious Diseases, Dr. Von Hauner Children’s Hospital, University Hospital, Ludwig-Maximilians-University Munich, Munich, Germany; 4 German Centre for Infection Research, PDZIF Partner Site Munich, Munich, Germany; 5 Institute of Infection, Veterinary and Ecological Sciences Liverpool, University of Liverpool, Liverpool, UK; 6 Alder Hey Children’s NHS Foundation Trust, Liverpool, UK; 7 Second Department of Paediatrics, National and Kapodistrian University of Athens, P. and A. Kyriakou Children’s Hospital, Athens, Greece; 8 Paediatric Immunology, Infectious Diseases & Allergy, Great North Children’s Hospital, Newcastle Upon Tyne Hospitals NHS Foundation Trust, Newcastle upon Tyne, UK; 9 Translational and Clinical Research Institute, Newcastle University, Newcastle upon Tyne, UK; 10 NIHR Newcastle Biomedical Research Centre Based at Newcastle Upon Tyne Hospitals NHS Trust and Newcastle University, Westgate Rd, Newcastle upon Tyne, NE4 5PL UK; 11 Section of Paediatric Infectious Diseases, Laboratory of Medical Immunology, Radboud Center for Infectious Diseases, Radboud Institute for Molecular Life Sciences, RadboudUMC, Nijmegen, the Netherlands; 12 Paediatric Infectious Diseases and Immunology, Amalia Children’s Hospital, RadboudUMC, Nijmegen, the Netherlands; 13 Paediatric Infectious Diseases and Immunology, Wilhelmina Children’s Hospital, University Medical Centre Utrecht, Utrecht, The Netherlands; 14 Section of Paediatric Infectious Diseases, Imperial College, London, UK; 15 Department of General Paediatrics, Medical University of Graz, Graz, Austria; 16 Department of Medicine, Population Health Sciences Institute, Newcastle University, Newcastle upon Tyne, UK; 17 Paediatric Emergency Medicine, Imperial College Healthcare Trust NHS, London, UK; 18 Vaccines, Infections and Paediatrics Research Group (GENVIP), Hospital Clínico Universitario de Santiago de Compostela. Genetics, Santiago de Compostela, Spain; 19 Department of Infectious Diseases and Faculty of Medicine, University Medical Centre Ljubljana, University of Ljubljana, Ljubljana, Slovenia; 20 London School of Hygiene and Tropical Medicine, Faculty of Tropical and Infectious Disease, London, UK; 21 Rīgas Stradiņa universitāte, Department of Paediatrics, Children Clinical University Hospital, Riga, Latvia

**Correction to: European Journal of Pediatrics** **https://doi.org/10.1007/s00431-022-04606-5**

In the original published version of the above article, Table 2 was incorrectly presented. The data is now presented correctly below:

**Table 2:** Patient characteristics and management (*N* = 913)

	**Febrile Children below 3 months**	**Range per ED** (%)	**Missing**
**Age** (months)^a^	1·7 (1·0–2·3)	0·9–2·3	-
**Gender** (boys)	526 (58)	(42–71)	-
**Referred**	495 (54)	(14–100)	10 (1)
**Triage urgency**			56 (6)
-Low	371 (41)	(4–96)	
-Intermediate/high	486 (53)	(4–87)	
**Ill appearance**	214 (23)	(4–57)	69 (8)
**Presenting symptom**^b^			
-Neurological	24 (3)	(0–13)	94 (10)
-Respiratory	438 (48)	(21–83)	143 (16)
-Gastrointestinal	201 (22)	(8–37)	137(15)
-Other	369 (40)	(9–62)	0 (0)
**Vital signs**			
Tachycardia	220 (24)	(6–46)	73 (8)
Tachypnea	176 (19)	(4–29)	193 (21)
Hypoxia	35 (4)	(0–9)	103 (11)
Temperature (°C)^a^	37·6 (37·0–38·2)	(37·1–38·5)	68 (7)
**Duration of fever** (days)^a^	0·5 (0·5–1·5)	0·5–0·5	88 (10)
**Increased work of breathing**	103 (11)	(1–21)	153 (17)
**Focus of infection**			-
-Upper respiratory tract	229 (25)	(3–50)	
-Lower respiratory tract	138 (15)	(0–30)	
-Gastrointestinal tract	54 (6)	(0–18)	
-Urinary tract	94 (10)	(4–24)	
-Flu like illness or exanthema	35 (4)	(1–10)	
-Soft tissue, skin or musculoskeletal	22 (2)	(0–7)	
-Sepsis/meningitis	81 (9)	(0–36)	
-Other	260 (29)	(9–46)	
**Diagnostic tests**			-
-**No diagnostic tests**	**179 (20)**	**(0**–**34)**	
-**Only simple diagnostic tests**^**c**^	**333 (37)**	**(16**–**83)**	
-*CRP*	*251 (75)*	*(27*–*100)*	
-*White blood cell count*	*242 (73)*	*(32*–*100)*	
-*PCT*	*10 (3)*	*(0*–*33)*	
-*Urinalysis*	*190 (57)*	*(23*–*80)*	
-*Urine culture*	*68 (20)*	*(0*–*67)*	
-*Ultrasound*	*26 (8)*	*(0*–*16)*	
-*Chest X-ray*	*46 (14)*	*(0*–*37)*	
-*Respiratory test/sputum culture*	*81 (24)*	*(7*–*60)*	
-**Advanced diagnostic tests**^**c**^	**401 (44)**	**(14**–**67)**	
-*CRP*	*394 (98)*	*(88*–*100)*	
-*White blood cell count*	*390 (97)*	*(81*–*100)*	
-*PCT*	*50 (13)*	*(0*–*92)*	
-*Urinalysis*	*293 (73)*	*(50*–*98)*	
-*Urine culture*	*288 (72)*	*(24*–*96)*	
-*Ultrasound*	*61 (15)*	*(0*–*31)*	
-*Chest X-ray*	*65 (16)*	*(0*–*50)*	
-*Respiratory test/sputum culture*	*152 (38)*	*(0*–*81)*	
-*Blood culture*	*392 (98)*	*(13*–*67)*	
-*Lumbar puncture*	*197 (49)*	*(0*–*38)*	
-*CT scan*	*3 (1)*	*(0*–*4)*	
-*MRI scan*	*0 (0)*	*(0)*	
**Antibiotic treatment**^cd^	**374 (41)**	**(23**–**54)**	-
-Oral	27 (7)	(0–45)	
-Parenteral	345 (92)	(55–100)	
-Narrow spectrum	33 (9)	(0–19)	
-Broad spectrum	334 (89)	(81–100)	
**Hospital admission**^ce^	**621 (68)**	**(34–86)**	-
-Hospital admission > 24h^d^	498 (80)	(17–74)	
-Admission to PICU	11 (2)	(0–7)	

Absolute numbers and percentages (%) are shown

^a^Median and interquartile range (IQR) are shown; range per ED: lowest and highest median are shown

^b^It is possible to have more than one presenting symptom. If children had no neurological, respiratory, and gastrointestinal symptoms, they were classified as other

^c^Percentages per subcategory are based on the total number of children per management category

^d^Missing data on route of administration antibiotics and type of antibiotics (0·5–2%), and duration of hospital admission (3%)

^e^No children died in this cohort

The original article has been corrected.

